# Pilot Study on the Cost of Some Oncohematology Diseases in Bulgaria

**DOI:** 10.3389/fpubh.2019.00070

**Published:** 2019-03-29

**Authors:** Konstantin Tachkov, Maria Kamusheva, Konstantin Mitov, Miglena Doneva, Guenka Petrova

**Affiliations:** Department of Organisation and Economy of Pharmacy, Faculty of Pharmacy, Medical University of Sofia, Sofia, Bulgaria

**Keywords:** cost of diseases, oncohematology, macro costing, copayment, patients cost

## Abstract

The goal of the current study is to perform a pilot study of the cost of some oncohematology diseases in Bulgaria. This is a pilot broader burden of disease research. The official report of the National health insurance fund provided information about the total expenditures paid for medicines, ambulatory services, and hospitalizations in 2015 and 2016. To evaluate the costs from a patient perspective, an internet inquiry was organized with the support of the patient organization. The inquiry contained questions regarding the patients' demography, type of oncohematology disease, year of diagnosis, quality of life (EuroQol v5D), and additional out of pocket expenditures. Quality of Life data were statistically analyzed and Kruskal-Wallis analysis of variance was performed. From 2015 to 2016 the number of patients with oncohematological diseases decreased by approximately 3000 people. Less than 30% were hospitalized and the hospitalization cost decreased, but the cost for medicines increased by nearly 1.5 million Euros. Cost for medicines almost tripled the hospitalization cost. The reported mean quality of life was 0.749 (SD 0.203). There was positive correlation between QoL and current disease state (*p* = 0.008) and age (*p* = 0.025). 42% reported to have additional expenditures related to their oncohematology disease, 22% reported other expenditures (diet, change of everyday habits etc.) and 42% reported to have productivity loses due to loss of employment or change of work, 44% of the respondents reported additional payment for medicines for concomitant diseases. Thus, the total cost (public funds and patients) accounted for 37,708,764 Euro. Despite the high public expenditures, the indirect costs due to productivity loses are higher. Costs for medicines are higher than costs of inpatient treatment, but this tendency is observed in all European countries. The increases in the costs of medicines are compensated by reduced costs of hospitalization. Despite their higher costs, newer medicines are an effective and reasonable investment from a societal perspective. Currently the higher levels of copayment increase the burden on the patients.

## Introduction

Treatment of hematological oncologic diseases has dramatically improved in the last decade, and the overall-survival of patients has seen an increase due to this (1–3).

Malignant tumors of the blood are a heterogeneous group of systemic disorders (4, 5). Of these, the most common and prevalent are following lymphoid lines: Hodgkin lymphomas, B-cell and T/NK cell lymphomas, acute or chronic lymphocytic leukemias, and plasma cell neoplasms (plasma cell myeloma included). Other common myeloid diseases include acute and chronic myeloid leukemia, myelodysplastic syndrome, and myeloid proliferative disorders.

The advances in molecular diagnostics methods and personalized medicine has allowed for the development of precision therapeutic schemes for each individual thus allowing physicians to achieve the therapeutic goals needed to alter the course of the disease (6, 7). The progress made in treating malignant hematologic diseases is undisputed, and due in most parts to the entry of multiple medicinal products into the market (8–14). Nonetheless, these new products constitute a financial burden on public funds, and necessitate estimating the cost-effectiveness of treating malignant diseases with these products (15–18). In Bulgaria, costs for onco-hematologic diseases are covered by the National health insurance fund (NHIF), but through separate tariff pathways for medicines, hospital stay, and clinical testing, respectively. Although, clinical tests and examinations of outpatients are also reimbursed, there is a limit on the number of tests per year. Some of the medicines for concomitant diseases are partly reimbursed and patients have to copay (19).

National studies in Bulgaria, which estimate the cost and outcomes of malignant hematological disease are limited, which prompted our interest to conduct this research (20, 21).

The goal of the current study is to perform a pilot study of the cost of some oncohematology diseases in Bulgaria.

## Materials and Methods

This is a pilot broader burden of disease research. An official report by the National health insurance fund provided information about the expenditures paid for onco-hematological diseases in 2015–2016 (22). Cost for medicines, ambulatory services, and hospitalizations were summed to obtain the total costs for each disease. Hospitalization costs have different health insurance tariffs and were calculated by multiplying the unit tariff cost for a particular diagnosis by the number of hospitalized patients (23).

To evaluate the costs from a patient perspective, an internet inquiry was organized with the support of the patient organization. The inquiry contained questions regarding the patients' demographics, type of oncohematology disease, year of diagnosis, quality of life (QoL), and additional out of pocket expenditures. Only patient reported onco-hematology diseases were included in the analysis and those were: Hodgkin and non-Hodgkin lymphoma, chronic lymphoid leukemia, chronic lymphatic leukemia, and other malignant lymphomas.

The quality of life was accesses via the standardized questionnaire EuroQol v5D (EQ5D/3L). EQ5D measures the quality of life (QoL) through five main domains that are mobility, self-care, usual activities, pain/discomfort, and anxiety/depression. Every domain is characterized with 3 levels of answers: no problem; moderate problems; severe problems. The scoring of each combinations of answers is used to evaluate the overall QoL from 0 (death) to 1 (perfect health). The questionnaire was tested, corrected and then distributed electronically among members of patient organizations. 109 patients responded and 97 with only onclohematologic diseases were included in the analysis.

Statistical analysis was conducted with MedCalc software. A Kurskal-Wallis non-parametric analysis was performed, since not all EQ5D data had a normal distribution and the following variables were tested for significant differences QoL and: age, gender, disease duration, current disease state, and type of disease. Median values are displayed for all tested parameters, along with the 25 and 75th percentile of the Inter-Quartile Range (IQR).

Indirect costs were calculated as productivity loses by first determining the proportion of people who reported losing work due to the disease then multiplying their percentage by the minimal monthly salary and calculated for 11 year.

## Results

During 2015-2016 the number of patients with oncohematological diseases in the country decreased by approximately 3,000 people. Less than half were reported by the NHIF to have used health care services and <30% had been hospitalized. The hospitalization cost decreased, but the cost for medicines increased by nearly 1.3 million Euros (Table 1). Cost for medicines almost tripled the hospitalization cost. These are primarily costs paid for ambulatory care of oncohematology patients. Hospitalization costs include the inpatient medicines and all inpatient health care services as part of the agreed NHIF tariffs. An increase of cost for medicines by 12.15% and decrease in number of patients by nearly 7.87% was observed in 2016 in comparison with 2015 (Table 1).

**Table 1:** Number of patients and costs for oncohematology patients paid by the health insurance fund.

**Indicator**	**2015**	**2016**
Number of patients with malignant oncohematology diseases	41,946	38,645
Number of hospitalized patients	12,171	10,913
Number of patients reported to use health care services (hospitalized plus outpatient)	17,087	16,076
Weighed Cost of hospitalizations (Euro)	4,214,473	3,778,863
Cost for medicines for ambulatory therapy (Euro)	10,558,344	11,841,477

### Results From the Inquiry and Costs Paid by the Patients

Patients' responses to the electronic questionnaire, which was distributed, are shown on Table 2.

**Table 2:** Characteristics of the responded patients and results from Kruskal-Wallis.

**Indicator**	**Number**	**Percent**	**Median QoL (25–75th percentile)**
**GENDER**			
- Male	38	38	0.704 (0.621–0.823)
- Female	59	62	0.704 (6.11–1.00)
Significance level			*P* = 0.744
**AGE GROUP**			
1–19	3	3	0.704 (0.356–0.704)
20–30	12	12	0.716 (0.704–1.00)
31–40	29	30	0.782 (0.621–1.00)
41–50	19	20	0.704 (0.614–0.716)
51–60	26	27	0.718 (0.624–1.00)
Above 60	8	8	0.553 (0.361–0.634)
Significance level			*P* = 0.025
**TYPE OF DIAGNOSIS**			
- Hodgkin lymphoma	39	40	0.716 (0.704–1.00)
- Non-Hodgkin lymphoma	31	32	0.704 (0.614–1.00)
- Chronic myeloid leukemia	4	4	0.587 (0.507–0.812)
- Chronic lymphatic leukemia	10	10%	0.710 (0.535–0.764)
- Other	13	13	0.625 (0.546–0.712)
Significance level			*P* = 0.075
**LENGTH OF LIFE WITH THE DISEASE**			
- Below 1 year	11	11	0.641 (0.611–0.713)
- 1–5 years	64	66	0.704 (0.624–1.00)
- Above 5 years	22	21	0.749 (0.549–1.00)
Significance level			*P* = 0.389
**CURRENT STATE**			
- Remission since 1 year	8	8	1.00 (0.666–1.00)
- Remission above 1 year	32	33	1.00 (0.666–1.00)
- Remission after recurrence	2	2	0.852 (0.704–1.00)
- Active treatment	46	47%	0.704 (0.611–0.716)
- Recurrence	8	8%	0.580 (0.535–0.665)
Significance level			*P* = 0.0089
**PRELIMINARY KNOWLEDGE ABOUT THE DISEASE**			
Yes	64	66	
No	17	18	
Type of cancer	16	16	
**CURRENT THERAPY**			
- Under monitoring	49	51	
- Transplantation	2	2	
- Chemotherapy	21	22	
- Target therapy	5	5	
- Other medicines	15	15	
- Other	3	5	
**PREVIOUS THERAPY**			
- Under monitoring	14	15	
- Transplantation	1	1	
- Chemotherapy	37	38	
- Target therapy	–	–	
- Other medicines	6	6	
- Other	6	6	
- Two types of therapy	23	24	
- Three types of therapy	5	5	
- Missing data	5	5	
**QUALITY OF LIFE**			
EQ5D/3L (average and SD)	0.749 (*SD* = 0.203)		

Among the respondents the majority were women (62%), most above 40 years of age (55%). Two-thirds of responders had Hodgkin and non-Hodgkin lymphomas (72%), and 89% had had the disease for over 1 year. Half of the patients were receiving active treatment, and 8% had a recurrence. The rest (42%) were in remission. 5 patients were treated with target therapy, while 21 were on other therapies.

The mean reported quality of life for all patients is 0.749 (SD 0.203), which is a relatively high value with low standard deviation (SD), pointing toward the homogeneity of answers. The reported disease duration and current disease state points toward high disease burden for patients–taking time from their life, a long period to recover, and requiring complicated therapy and monitoring. Despite this, the QoL in patients is relatively high, indicating good control, which could be explained by a number of innovative medicines implemented in the practice and improved medical care for the affected.

The majority of costs were covered by the NHIF. 42% of patients reported to have additional expenditures related to their oncohematology disease; 22% reported other expenditures (diet, change of everyday habits etc.) and 42% reported to have productivity loses due to loss of employment.

Only 44% of the respondents reported co-payment for medicines for concomitant disease, median 150 euro (95% CI−116.95–200.00). Copayment for hospitalization was reported by 5%, $$ and 5% replied that they co-paid for clinical tests and consumables. Most often this was echography, nuclear magnetic resonance, kits for testing, and copayment varied between 50 and 150 euro Median 100.00 euro (95% CI 95.00–150.00 euro).

### Total Cost of Therapy

**Figure 1:**
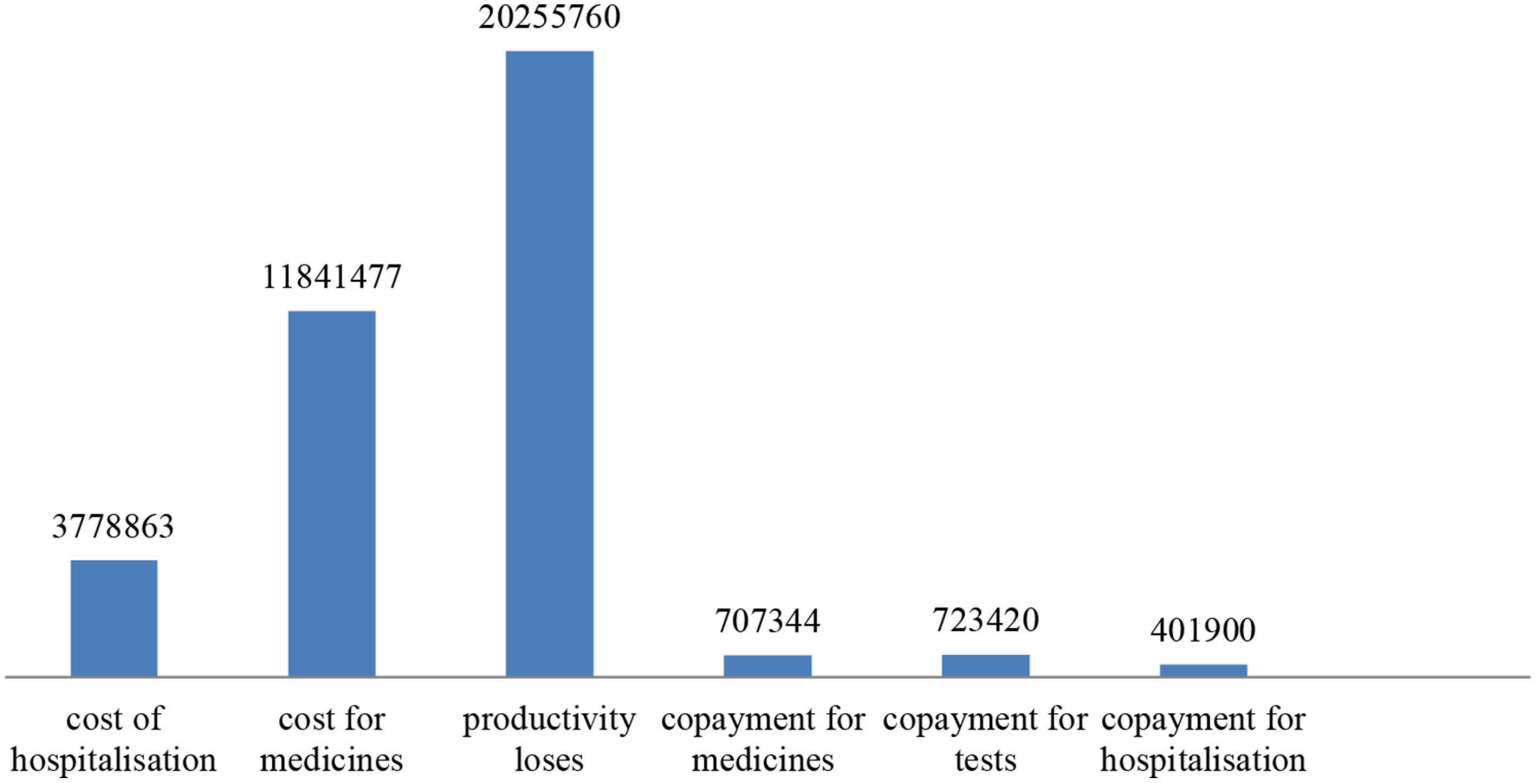
Total costs for oncohematology diseases.

If we apply the distribution obtained via the responses to the total number of 16 076 oncohematology patients that reported to use health care services by the NHIF, we estimate that 6752 patients will experience loss of productivity (42%). Assuming they all earn the minimal wage for the country −250 euro, this will amount to 20,255,760 Euro for 1 year. Copayment for medicines will amount to 707,344 euro (44% out of 16076 paid per 100 euro during year). For hospitalizations was paid 401,900 euro for all patients for 1 year and for tests and consumables 723,420 Euro additional payment. Thus, the total cost (public funds and patients) account for 37,708,764 Euro. The structure of the cost is shown in Figure 1.

Despite the high expenditures by public funds, the indirect costs due to productivity loses are higher and together with copayments from the patients exceed the public expenditures.

### Statistical Analysis

Neither the patients' gender nor the type of disease had a statistically significant influence on their quality of life (*p* = 0.74 and *p* = 0.07, respectively). The lowest QoL values were observed in patients with HLL and those with “other” leukemia's. These results could likely be due to the different subtypes of the different diseases.

A statistically significant difference was observed between quality of life and age (*p* = 0.025). Patients over the age of 60 had the lowest median quality of life (0.553), a statistically significant difference from all other age groups. These results are not surprising, considering that, because of advances in treatment, oncohematologic disease have a chronic nature. Elderly patients have had to live with the disease for a longer duration which, in combination with concomitant diseases, negatively impacts quality of life.

Current status of disease also significantly affects the quality of life (*p* = 0.0089); naturally the highest QoL values were observed in patients, who were in remission. The highest burden was on patients who experienced a remission, where the analysis of difference revealed that it was statistically significant from patients who were in remission. The *post-hoc* test also showed a statistically significant difference between patients who were undergoing active treatment, compared to those in remission (Table 3). These results confirm that the current state of the disease carries a burden on patients. Although the result did not reach statistical significance, the type of disease also registered an effect on quality of life values. Patients with chronic myeloid leukemia reported the lowest median values (0.587).

**Table 3:** *Post-hoc* analysis of difference between current state of disease and QoL data.

**Factor**	***n***	**Average rank**	**Different (*P* < 0.05) from factor nr**
1. Remission up to 1 year	8	60.00	(5)
2. Remission for over 1 year	32	58.38	(4) (5)
3. Remission after recurrence	2	62.75	
4. Active treatment	46	42.91	(2)
5. Recurrene	8	26.06	(1) (2)
Degrees of freedom (DF)	4		
Significance level	*P* = 0.008929		

## Discussion

Our results in this study are rather positive in terms of quality of life and other patient characteristics. However, one major consideration should be that online questionnaires introduce a certain bias when estimating QoL. Only patients who are in a stable state or they have already achieved good disease control and are recovering are likely to respond. Because of this, the quality of life reported in this study has higher values than expected. Similar results have been reported in other studies and they support our findings that there is a link between quality of life, age, and state of disease (24, 25).

Copayment for health services is mostly required for additional tests (both diagnostic and prophylactic), while they are rarely required for hospital stay and inpatient medicines therapy. Despite this, when putting together costs covered by the NHIF and those paid by patients, it became clear that the majority of the costs were covered by the patients out of pocket thus they experienced a higher burden. The newest diagnostic methods and testing kits are, as of the moment of writing this article, not covered by the NHIF, which leads to patients experiencing an added financial burden (22).

In 2016 a systematic review of the costs associated with malignant diseases in Europe was published in The Lancet Hematology (21). The study, which was conducted in 2012, included also data from Bulgaria, which the authors had obtained from international sources. The research presented results from 28 European countries plus Iceland, Norway and Switzerland, Greece and other South East European countries. The authors discovered that for 2012 the total costs associated with blood disorders amounted to €23 billion ($25 billion), whereby half of these costs were attributable to malignant oncohematology diseases such as Hodgkin's lymphoma, non-Hodgkin lymphomas, multiple myeloma, and leukemia. Malignant oncologic diseases cost €12 billion ($13 billion), with 60% of the costs attributable to health services, and only a third of them for medicines (21). When reviewing the article, we summarized that the costs associated with health services amounted to €7.3 billion, while indirect costs due to loss of productivity amounted to €3.6 billion (30%), and informal care was €1 billion. To put this in context, Bulgaria scores in second place for lowest costs, right behind Lithuania. The authors further calculated that in Europe the number of working days lost, due to premature death, amounted to 90 000 which equates to 2 billion Euro (16% of total indirect costs), whereas the total number of days missed from work amounted to 12 million days (14% of total costs). These data were not available for Bulgaria. Other costs, which were not included for the country, were costs for primary care, emergency care, and emergency examinations.

The authors of this systematic review estimated that the costs for Bulgaria were 19,198, 000 Euro, well-lower than the costs we estimated for 2016–approximately 34.5 million Euro. Contrary to our own calculations, those losses due to premature death were calculated to be 9.1 million Euro and those due to loss of productivity 4.3 million euro, which are slightly lower numbers than the ones we calculated. This discrepancy could be due to the difference in methodology when calculating indirect costs. It is evident that Bulgaria generates low costs for malignant hematologic diseases compared to other European countries included in this study.

In general, the access to modern onco-hematology therapies is limited that has been also reported by other studies from the region (26).

A limitation to our study is the use of an online questionnaire to estimate patient quality of life and extra costs. These types of questionnaires are usually answered by patients, who actively participate in their disease therapy and have achieved good disease control. Our results are very similar to the results of the cited systematic review, which is strong evidence as to the validity of our findings (21).

The other limitation of the study is the lack of any other structured data for oncohematology diseases expenditures by the NHIF from previous years. All costs were reported on total for all oncology diseases and there is no official information per diagnoses as started to be available since 2015. This fact limited the possibilities to observe a longer time period.

Our study observed decreasing costs for the National health insurance fund that could be explained by the strict expenditures control measures active in the country, as well as recent regulatory changes concerning methods of diagnosing and assigning active treatment to malignant diseases. The prescribing of new medicines requires permission of specialized physicians' committee and reevaluation after 6 months for its necessity on the basis of performed therapeutic results.

## Conclusion

Costs for medicines are higher than costs of inpatient treatment, but this tendency is observed in all European countries. The increases in the costs of medicines are compensated by reduced costs of hospitalization. Despite their higher costs, newer medicines are an effective and reasonable investment from a societal perspective. Currently the higher levels of copayment increase the burden on the patients.

## Data Availability

The datasets generated for this study are available on request to the corresponding author.

## Author Contributions

KT, MK, and GP made contributions to the conception, design, and drafting of the work. KM and MD performed the statistical analysis and interpretation. All authors revised the text and approved the final version of this manuscript.

### Conflict of Interest Statement

The authors declare that the research was conducted in the absence of any commercial or financial relationships that could be construed as a potential conflict of interest.
